# *Mdm2-P53* Interaction Inhibitor with Cisplatin Enhances Apoptosis in Colon and Prostate Cancer Cells *In-Vitro*

**DOI:** 10.31557/APJCP.2019.20.11.3341

**Published:** 2019

**Authors:** Amit Gupta, Tapan Behl, Hem Raj Heer, Rahul Deshmukh, Pyare Lal Sharma

**Affiliations:** 1 *Animal Tissue Culture Laboratory, Department of Pharmacology, Indo Soviet Friendship College of Pharmacy, *; 3 *Indo Soviet Friendship College of Pharmacy Moga, *; 2 *Department of Pharmacology, Chitkara Collge of Pharmacy, Chitkara University, Rajpura,*; 4 *Department of Pharmaceutical Sciences and Technology, Maharaja Ranjit Singh Punjab Technical University, Bathinda, Punjab, *; 5 *5Emeritus, Post Graduate Institute of Medical Education and Research, Chandigarh, India.*

**Keywords:** MDM2, p53 interaction, cisplastin, apoptosis, colon and prostate cancer cells

## Abstract

**Objective:**

To study the effect of RITA (*MDM2-p53* interaction inhibitor) and its action along with genotoxic drug cisplatin was evaluated on COLO-205 colon cancer and PC-3 prostate cancer cells.

**Method:**

Various *in-vitro* parameters to determine cytotoxic and apoptotic potential of RITA with genotoxic drug cisplatin were evaluated. The potentiation of cytotoxic effect was evaluated using MTT assay and colony forming assay, mechanism of cell death by Etbr/AcO assay and the mechanism of apoptosis was determined by caspase-3 release assay.

**Results:**

The findings from MTT confirmed the best possible potent combination of 5+5µM and 10+10µM concentration of Cisplatin and RITA respectively. These combinations were further evaluated for its chemo sensitizing effect which confirmed the significant reduction in number of colonies in combination as compared to monotherapy. Also, the results of Etbr/AcO assay were in line with the colony forming assay. For apoptotic activity, it was noted that increasing the concentration of cisplatin and RITA (10µM), did not affect much to apoptotic activity and was found to be equally effective to that of low dose (5µM) concentration. The same results were seen in Caspase-3 release effect on both the cell lines.

**Conclusion:**

Our present study provides compelling evidence that pharmacological activation of the p53 by blocking the MDM2–p53 interaction is a promising cancer therapeutic strategy and using RITA in combination with Cisplatin not only decrease the toxic effect of Cisplatin by decreasing its dose but also increasing the apoptotic effect, warrants clinical evaluation on both colon and prostate cancer.

## Introduction

The tumor suppressor *p53* plays a key role in the regulation of cell cycle, apoptosis, DNA repair, senescence (Teodoro et al., 2007; Fridman and Lowe, 2003; Vousden and Lu, 2002) and also known to suppress oncogenesis in humans (Petitjean et al., 2007). In several cancers, the gene encoding p53 is either deleted or mutated, rendering the p53 protein inactive (Suzuki and Matsubara, 2011; Soussi and B´eroud, 2011). In few cancers, *p53* retain as wild-type where its function is effectively inhibited by mouse double minute 2 (*MDM2*) oncoprotein, a primary cellular inhibitor. *MDM2* is a *p53*-specific E3 ubiquitin ligase that mediates the ubiquitin-dependent degradation of *p53* (Michael and Oren, 2003; Momand et al., 1998). In addition, *MDM2* binds to *p53* transactivation domain and suppress p53-mediated transcription (Schuler and Green, 2005; Schuler et al., 2003). Thus, *MDM2* expression is positively regulated by *p53* transactivation that forms a feedback loop. Amplified *MDM2* has been reported in human sarcomas (Oliner et al., 1992; Gunther et al., 2000) and in 10% of various human cancers including gastric carcinomas (Sun et al., 2004; Fojo, 2002). One attractive pharmacological approach to p53 reactivation is to use a small molecule to block the *MDM2–p53* interaction (Rippin et al., 2002; Reifenberger et al., 1993). 

Reactivation of *p53* and induction of tumor cell apoptosis (RITA) is a small molecule that inhibits the growth of wild-type *p53* colon carcinoma cells with minimal effect without wild-type p53 (Gu et al., 2006). RITA and nutlins binds to *MDM2*, block its interaction with p53, and activate wild-type *p53* (Vassilev et al., 2004; Zhao et al., 2010; Issaeva et al., 2004; Endo et al., 2011; Koster et al., 2011; Shinohara et al., 2007). RITA increases *p53* in human tumor cells that express wild-type *p53* (Issaeva et al., 2004). RITA alone has been found to have promising effects. In the present study, we evaluated the combination of RITA with the currently existing genotoxic drug. We tested colon and prostate cancer cell lines for the expression of *MDM2* and their sensitivity to RITA with anticancer drugs. We also investigated in vitro antitumor effects of RITA alone and its enhancement by cisplatin in human colon and prostate cancer cell lines with wild-type p53.

## Materials and Methos

Chemicals: 5, 5’-(2,5-Furandiyl) bis-2-thiophenemethanol, a known chemical for RITA was purchased from Tocris, USA. Cisplatin was a kind gift from Adley group. 

Cell lines and maintenance: Wild-type p53 cell lines viz. COLO-205 (Colon) and PC-3(Prostrate) were obtained from NCCS, India). The cell lines were maintained in RPMI-1640 and F-12 Ham media, respectively. Both cell lines were supplemented with 10% inactivated fetal bovine serum, 100U/ml penicillin and 100U/ml streptomycin incubated at 370C and 5% CO_2_ in humidifier incubator (New Brunswick, USA). After attaining 80% confluence cells were sub-cultured by trypsinization with 0.25% trypsin solution under sterile conditions.

*In vitro growth inhibition assay (MTT assay)*

Cytotoxic evaluation of drug solutions was done by tetrazolium-based colorimetric method using 3-(4,5-Dimethylthiazol-2-yl)-2,5-Diphenyltetrazolium Bromide (MTT) assay. Cells were plated in 96-well plates at 5×10^3^ per 100μl per well with density determined based on growth characteristics of each cell line. After overnight incubation, triplicate wells were treated with varying concentration of compounds ranging from (1-200µg/ml) and standard doxorubicin incubated for 3 days. After 3 days, medium was replaced with 2µl of MTT solution (5mg/ml) (Himedia, India) and cells were incubated for 3 hours. Formazan crystals were dissolved in dimethylsulfoxide (DMSO) (Himedia, India). The relative percentages of metabolically active cells compared with untreated controls were determined on the basis of mitochondrial conversion of MTT to formazan crystals dissolved in DMSO. Spectrophotometric absorbance of sample was determined by microplate reader (BIORAD) at 570/630nm.

*Chemo sensitizing effect of drug*

*Colony Forming Assay*

Colony forming assay is an effective method to measure the anti-cancer and anti-proliferative effects. Cells were grown in vitro in soft agar medium and highly viscous medium, methylcellulose. Semisolid media reduce the cell movement and allow individual cell to develop as colonies. Briefly, equal volume of 1% agarose and 2x RPMI and F-12 Ham medium (20%FBS) was warmed at 40ºC and added in each well of 6-well plate and allowed to gel. Approximately 4,000-8,000 cells were mixed with nutrient medium containing agarose and various compounds alone or in combination and seeded on top of the plate and incubated at 37ºC at 5% CO_2 _incubator for 10-15 days. Colonies were examined under microscope after staining with Giemsa’s staining solution (1%) after 16 days. 

*Apoptosis Study*

*EB/AO method*

Both COLO-205 and PC-3 cells were spinned at 1,000 RPM for 5 minutes and washed with 1 ml of cold PBS solution. Cell pellets were then re-suspended in 25μl cold PBS and 2μl EB/AO dye mix was added. Stained cell suspension (10μl) were placed on a clean microscope slide and covered with a cover slip. Cells were viewed and counted using an Olympus inverted microscope CK X41 at 400x (USA). Images were captured using Sony digital camera. Tests were done in triplicate, counting a minimum of 100 total cells each.

*Caspase-3 Assay*

Caspase-3 release was tested using Biovision caspase-3 assay kit. Cells were incubated with and without drug treatment in 6-well plate. Concurrently, control culture was incubated without induction. Cells were counted and pellet of 1-5 X 10^6^ cells was collected. Resuspended the cells in 50 µL of chilled Cell Lysis Buffer and incubate cells on ice for 10 minutes. Cells were then centrifuged for 1 min in a microcentrifuge (10,000 x g). Supernatant (cytosolic extract) was transferred to a fresh tube and put on ice for immediate assay or aliquot and store at -80°C for future use. Assay protein concentration was determined.50-200 µg protein to 50 µL Cell lysis buffer for each assay was diluted. 50 µL of 2X Reaction Buffer (containing 10mM DTT) to each sample was added. Added 5 µL of the 4mM DEVD-pNA substrate (200 µM final cone.) and incubated at 37°C for 1-2 hour. Samples were read at 400 or 405 nm in a microtiter plate reader (BIO-RAD).

## Results

*Effect of cisplatin and RITA on cell viability in COLO-205 cell lines*

**Figure 1:**
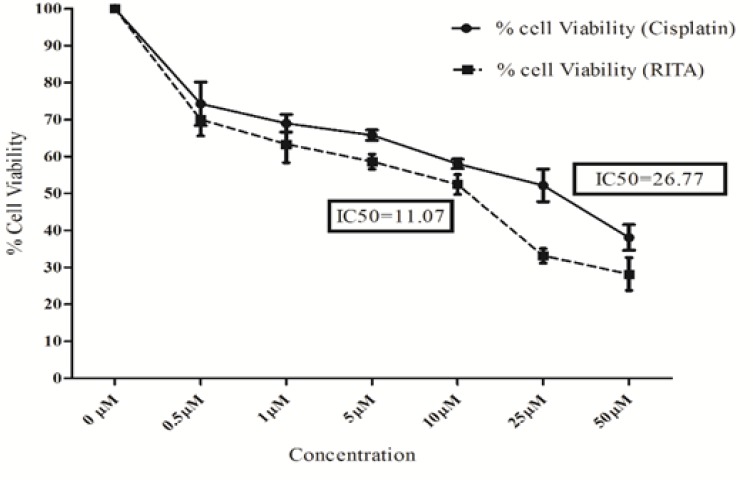
Effect of Cisplatin and RITA on Percentage Cell Viability in COLO-205. IC_50_ values are depicted alone for both cisplatin and RITA. Values are expressed as Mean **±**S.D

The effect of cisplatin and RITA on Viability of COLO-205 was checked using MTT assay. Both the drugs were able to reduce viability of COLO-205 in a dose dependent manner, as shown in Figure 1. After 72 hrs of treatment, both the drugs were found to be cytotoxic to colon cancer cell at the dose of 26.7µM and 11.0µM (and higher) respectively

*Effect of Cisplatin and RITA on cell viability in PC-3 cell lines*

**Figure 2:**
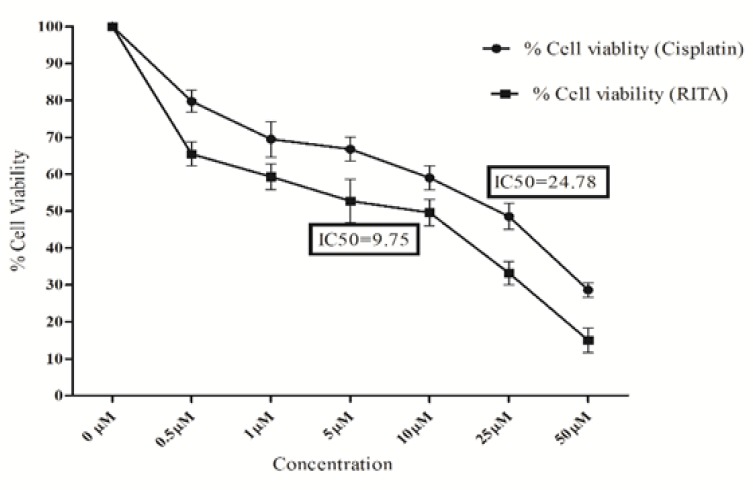
Effect of Cisplatin and RITA on Percentage Cell Viability in PC-3 Cells. IC_50_ values are depicted alone for both Cisplatin and RITA. Values are expressed as Mean **±**S.D

Further the effect of cisplatin and RITA on Viability of PC-3 cells was also checked using MTT assay. Both the drugs were able to reduce viability of PC-3 in a dose dependent manner, as shown in Figure 2. 

After 72 hrs treatment, both the drugs were found to be cytotoxic to COLO-205 at the dose of 24.8µM and 9.7µM (and higher) respectively.

*Selection of best combination of Cisplatin and RITA*

**Figure 3:**
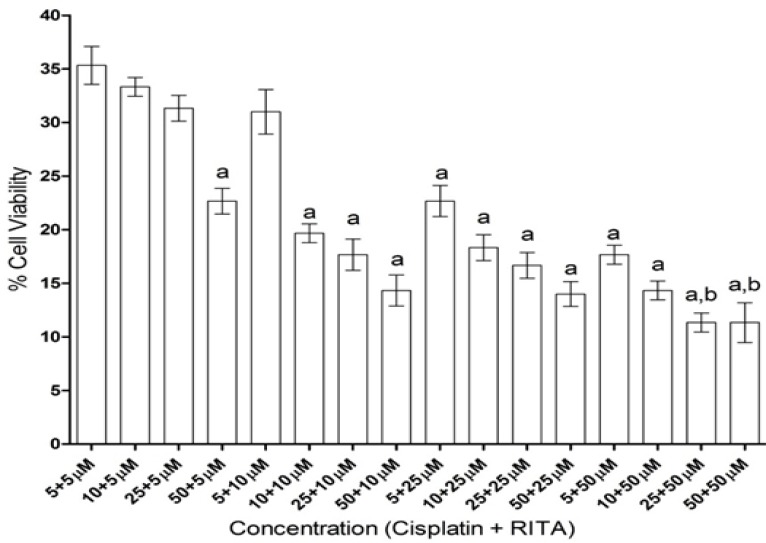
Effect on Percentage Cell Viability on COLO-205 by Using Various Concentrations of Cisplatin and RITA. Values are expressed as Mean **±**S.D. ^a^p<0.05 vs. 5+5µM Cisplatin-RITA combination, ^b^p< 0.05 vs. 10+10µM Cisplatin-RITA combination

Various combinations of cisplatin and RITA were analyzed on cell viability (Figure 3). Lowest toxic but effective dose of 5+5µM and high effective dose of 10+10µM were selected. Though the effective results came with the low dose itself, high dose selection was basically done to show that there is no dose-dependent effect of the drug. Whereas increasing the concentration of cisplatin and RITA (10µM), did not affect much to % cell viability and was found to be equally effective to that of low dose (5µM) concentration.

*Combination effect of Cisplatin and RITA alone and in combination on percentage cell viability on COLO-205*

**Figure 4:**
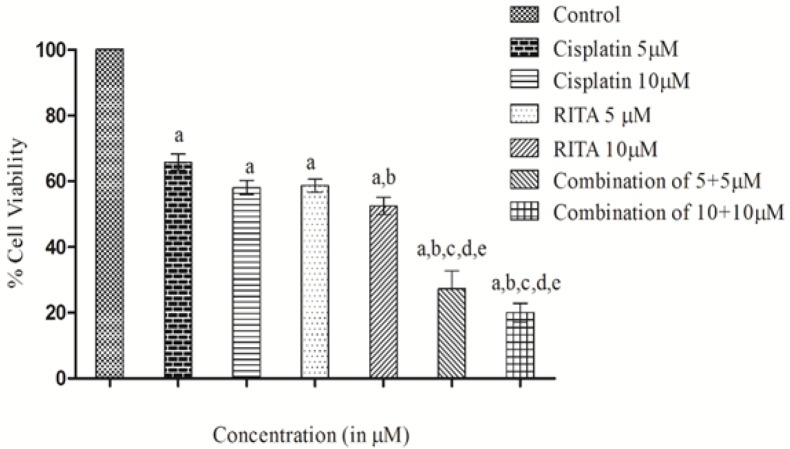
Effect of Cisplatin and RITA Alone and in Combination at 5+5µM and 10+10µM on Cell Viability in COLO-205 Cell Lines. Values are expressed as Mean **±** S.D. ^a^p<0.05 vs. control, ^b^p<0.05 vs. cisplatin 5µM, ^c^p<0.05 vs. cisplatin 10µM, ^d^p<0.05 vs. RITA 5µM, ^e^p<0.05 vs. RITA 10µM

Combination of Cisplatin and RITA was given at the dose of 5+5µM and 10+10µM as well as drugs alone on COLO-205 as shown in Figure 4. The combination of Cisplatin and RITA produced significant decreased the % cell viability compared with control, as well as their individual effect at 5µM and 10µM concentration. Whereas increasing the concentration of cisplatin and RITA (10µM), did not affect much to % cell viability and was found to be equally effective to that of low dose (5µM) concentration

*Combination effect of Cisplatin and RITA alone and in combination on percentage cell viability on COLO-205 and PC-3 cell lines*

**Figure 5:**
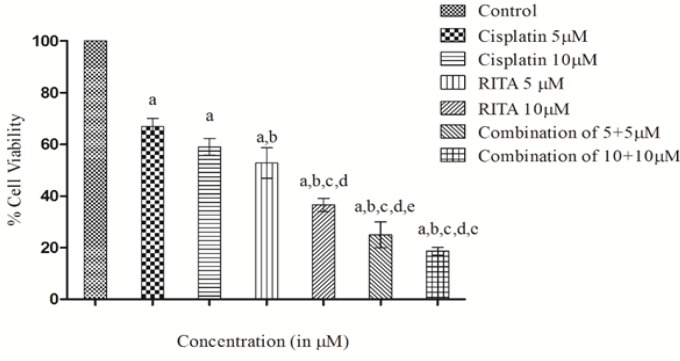
Effect of Cisplatin and RITA Alone and in Combination at 5+5µM and 10+10µM on cell viability in PC-3 cell lines. Values are expressed as Mean **±**S.D. ^a^p<0.05 vs. control, ^b^p<0.05 vs. cisplatin 5µM, ^c^p<0.05 vs. cisplatin 10µM, ^d^p<0.05 vs. RITA 5µM, ^e^p<0.05 vs. RITA 10µM

Combination of Cisplatin and RITA was given at the dose of 5+5µM and 10+10µM as well as drug alone on prostate cancer cell lines (PC-3) as shown in Figure 5. The combination of Cisplatin and RITA produced significant decreased the % cell viability compared with control, as well as their individual effect at 5µM and 10µM concentration. Whereas increasing the concentration of cisplatin and RITA (10µM), did not affect the % of cell viability and was found to be equally effective to that of low dose (5µM) concentration

*Chemo sensitizing effect of drug*

*Colony Forming Assay (CFA) on COLO-205*

**Figure 6:**
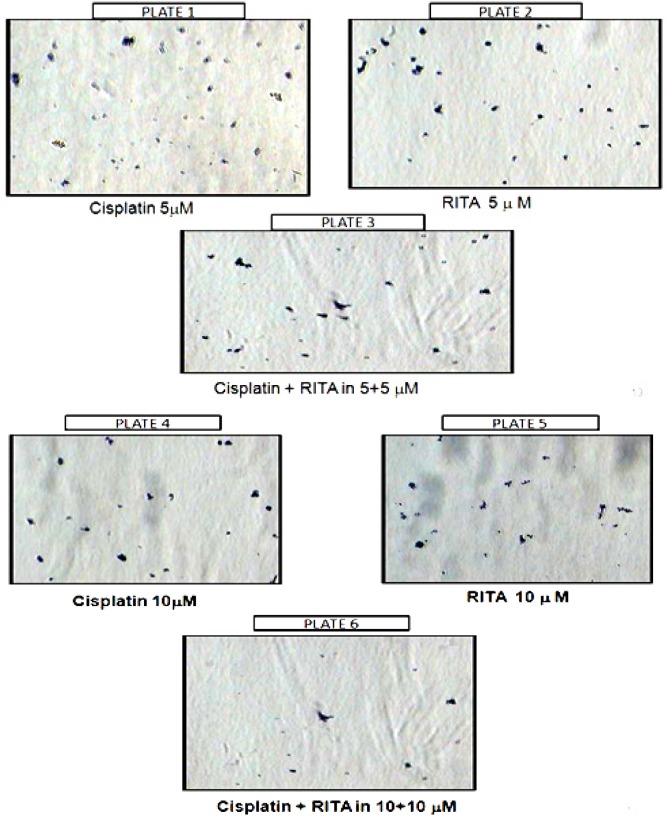
Effect of Cisplatin and RITA Alone and in Combination on Apoptosis on Colony Forming Assay in COLO-2015 Cell Lines

**Figure 7.**
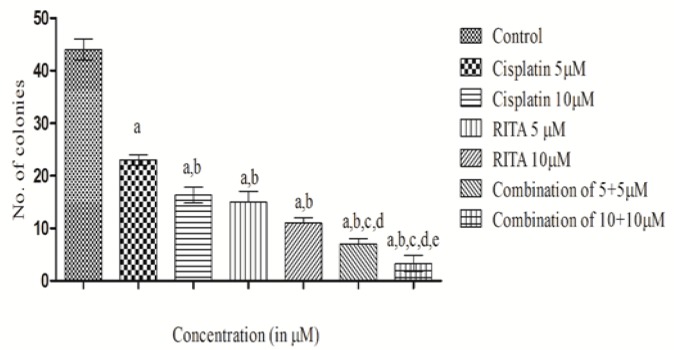
Effect of Cisplatin and RITA Alone and in Combination at the Dose of 5+5µM and 10+10µM on CFA in COLO-205. Values are expressed as Mean **±** S.D. ^a^ p<0.05 vs. control, ^b^ p<0.05 vs. cisplatin 5µM, ^c^ p<0.05 vs. cisplatin 10µM, ^d^ p<0.05 vs. RITA 5µM, ^e^ p<0.05 vs. RITA 10µM

To reconfirm the chemo sensitizing effect of the Cisplatin-RITA combination, we performed the CFA with COLO-205 colon cancer cells (Figure 6 and Figure 7). The results of CFA were corroborative results of the MTT assay. As can be visualized from (Figure 6), the combination of Cisplatin-RITA had almost negligible colonies. The chemo sensitizing effect of Cisplatin-RITA combination; and observed the significant reduction in number of colonies in combination of Cisplatin-RITA as compared to alone drugs. This proves that the Combination approach sensitized the cells to sub-toxic levels of cisplatin.

*Colony forming assay on PC-3 cells*

**Figure 8:**
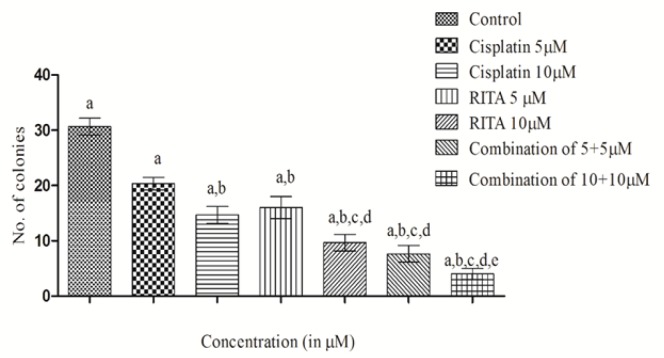
Effect of Cisplatin and RITA Alone and in at the Dose of 5+5µM and 10+10µM on CFA in PC-3 cells. Values are expressed as Mean **±**S.D. ^a^ p<0.05 vs. control, ^b^ p<0.05 vs. cisplatin 5µM, ^c^ p<0.05 vs. cisplatin 10µM, ^d^ p<0.05 vs. RITA 5µM, ^e^ p<0.05 vs. RITA 10µM

Similiarly, as can be visualized from Figure 8, the combination of Cisplatin-RITA had almost negligible colonies on PC-3 cell line to reconfirm the chemo sensitizing effect of Cisplatin-RITA combination; and observed the significant reduction in number of colonies in combination of Cisplatin-RITA as compared to alone drugs. This proves that the Combination approach sensitized the cells to sub-toxic levels of Cisplatin.

*Apoptotic assay*

*Acridine Orange/ Ethidium Bromide assay (Etbr/AcO) on COLO-205*

**Figure 9:**
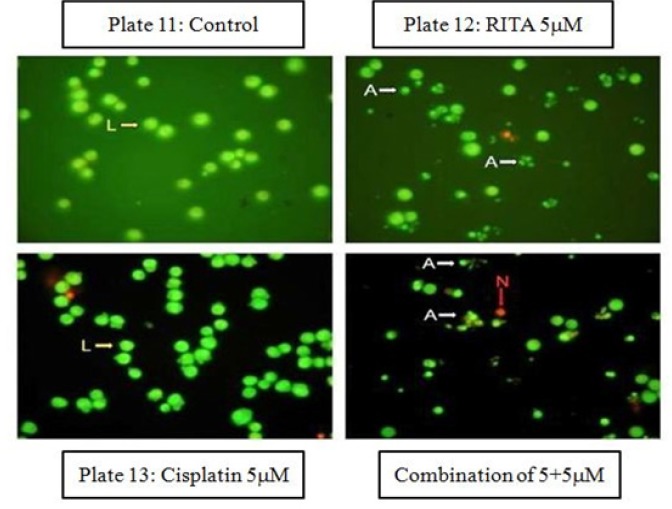
Effect of Drug after 24 hrs of Treatment in Etbr/AcO Assay. Magnification (100x) micrograps of COLO-205 shows that untreated cell nuclei are stained green and necrotic cells are stained red; whereas apoptotic cell are stained green with cell membrane ruptured with leakage of cytoplasm

**Figure 10:**
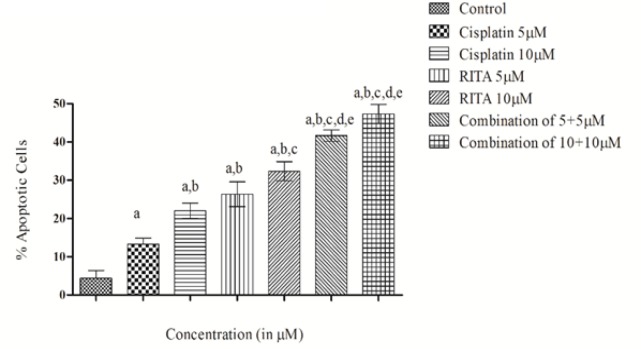
Effect of Cisplatin and RITA Alone and in Combination at the Dose of 5+5µM and 10+10µM on Etbr/AcO in COLO-205 cells. Values are expressed as Mean **±**S.D. ^a^ p<0.05 vs. control, ^b^ p<0.05 vs. cisplatin 5µM, ^c^ p<0.05 vs. cisplatin 10µM, ^d^ p<0.05 vs. RITA 5µM, ^e^ p<0.05 vs. RITA 10µM

**Figure 11:**
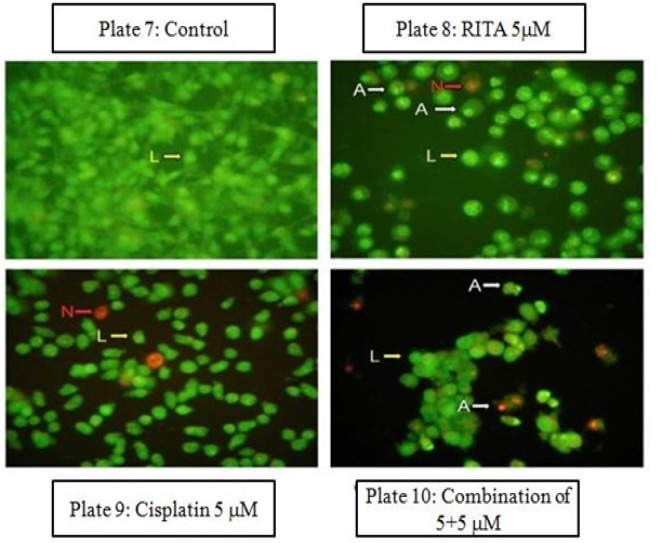
Effect of Drug after 24 hrs of Treatment in Etbr/AcO Aasay. Magnification (100x) micrographs of PC-3 shows that untreated cell nuclei are stained green and necrotic cells are stained red; whereas apoptotic cell are stained green with cell membrane ruptured with leakage of cytoplasm

**Figure 12.**
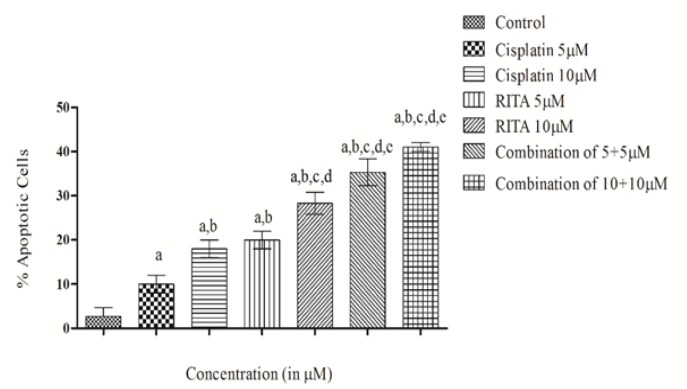
Effect of Cisplatin and RITA Alone and in Combination at the Dose of 5+5µM and 10+10µM on Etbr/AcO in PC-3 Cells. Values are expressed as Mean **±** S.D. ^d^ p<0.05 vs. Control, ^e^ p<0.05 vs. cisplatin 10µM, ^f^ p<0.05 vs. cisplatin 10µM, RITA 10µM

The induction of apoptosis was observed by Acridine Orange/Ethidium Bromide double staining of treated cell nuclei with optimized dose of Cisplatin, RITA, and their combination. Microscopic images of the dual stained cells, presented in Figure 9 and Figure 11, shows that the live cells nuclei stained green due to Acridine orange uptake and dead cells appear red due to Ethidium Bromide. Cells treated with combination of both drugs shows significant increase in apoptotic activity as compared to alone drug in COLO-205 (Figure 11). Figure 10 and Figure 12 clearly depicts that the combination approach induces apoptosis far more as compared to alone drugs. Combination of Cisplatin and RITA was given at the dose of 5+5µM and 10+10µM as well as drug alone on prostate cancer cell lines as shown in Figure 11 and Figure 12. The combination of Cisplatin and RITA produced significant increase in apoptotic activity as compared with control, as well as their individual effect at 5µM and 10µM concentration. Whereas increasing the concentration of cisplatin and RITA (10µM), did not affect much to apoptotic activity and was found to be equally effective to that of low dose (5µM) concentration. 

*Acridine Orange/ Ethidium Bromide assay (Etbr/AcO) on PC-3*

Microscopic images of the dual stained cells, presented in Figure 11, shows that the live cells nuclei stained green due to Acridine orange uptake and dead cells appear red due to Ethidium Bromide. Cells treated with combination of both drugs shows significant increase in apoptotic activity as compared to alone drug in PC-3 cells. Cells treated with combination of both drugs shows significant increase in apoptotic activity as compared to alone drug in PC-3. Figure 12 clearly depicts that the combination approach induces apoptosis far more as compared to alone drugs. Combination of Cisplatin and RITA was given at the dose of 5+5µM and 10+10µM as well as drug alone on prostate cancer cell lines . The combination of Cisplatin and RITA produced significant increase in apoptotic activity as compared with control, as well as their individual effect at 5µM and 10µM concentration. Whereas increasing the concentration of cisplatin and RITA (10µM), did not affect much to apoptotic activity and was found to be equally effective to that of low dose (5µM) concentration. 

*Caspases-3 Release effect on COLO-205*

**Figure 13:**
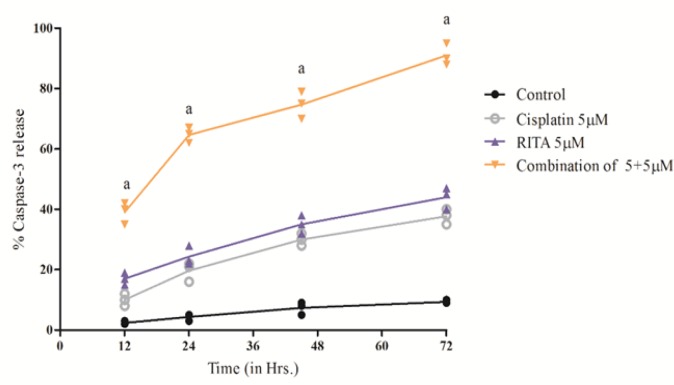
Effect of Cisplatin and RITA Alone and in Combination on the Caspase-3 Release on COLO-205. Caspase activity was determined after 12, 24, 48, 72 hrs of treatment. Values are expressed as Mean **±** S.D. ^a^ p<0.05 vs. Control, Cisplatin 5µM, RITA 5µM

**Figure 14:**
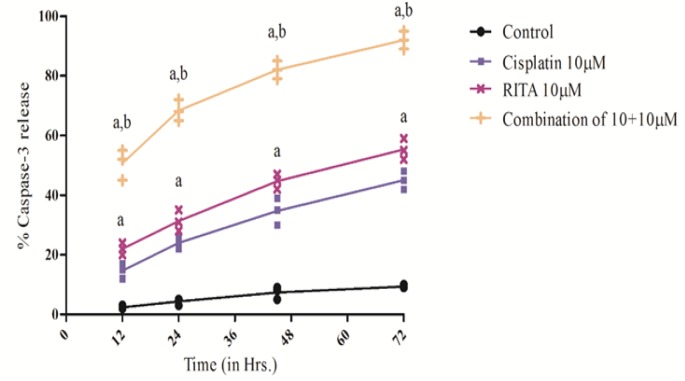
Effect of Cisplatin and RITA Alone and in Combination on the Caspase-3 Release on COLO-205. Caspase-3 activity was determined after 12, 24, 48, 72 hrs of treatment. Values are expressed as Mean **±** S.D. ^a^ p<0.05 vs. Control, ^b^ p<0.05 vs. Cisplatin 10µM, RITA 10µM

On getting remarkable results using Etbr/AcO assay, the molecular pathway involved in apoptosis was determined. Caspase-3 activity was determined after 12, 24, 48, 72 hrs of treatment. Our result demonstrated that the Combination enhances the degree of caspase-3 release as compared to the alone drugs and release of caspase-3 was also significantly increased from 12 hrs to 72 hrs of treatment as shown in Figure 13 and Figure 14. The combination of Cisplatin and RITA significantly increased the caspase-3 release as compared with control, as well as their individual effect at 5µM and 10µM concentration. Whereas increasing the concentration of cisplatin and RITA (10µM), did not affect much to caspase-3 release and was found to be equally effective to that of low dose (5µM) concentration.

*Casapases-3 Release effect on PC-3*

**Figure 15:**
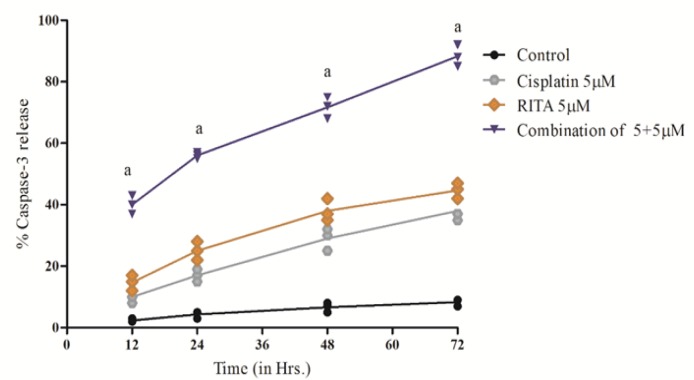
Effect of Cisplatin and RITA Alone and in Combination (5+5µM) on the Caspase-3 Release on PC-3 Cells. Caspase activity was determined after 12, 24, 48, 72 hrs of treatment. Values are expressed as Mean **±** S.D. ^a^ p<0.05 vs. Control, Cisplatin 5µM, RITA 5µM

**Figure 16:**
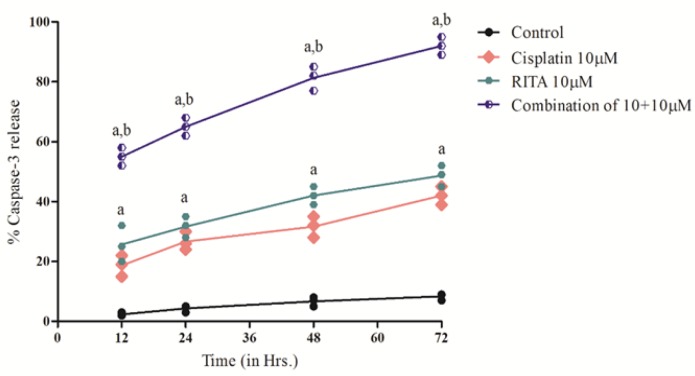
Effect of Cisplatin and RITA Alone and in Combination (10+10µM) on the Caspase-3 release on PC-3 Cells. Caspase activity was determined after 12, 24, 48, 72 hrs of treatment. Values are expressed as Mean **±** S.D. ^a^p<0.05 vs. Control, ^b^ p<0.05 vs. Cisplatin 10µM, RITA 10µM

Caspase-3 activity was determined after 12, 24, 48, 72 hrs of treatment. Our result demonstrated that the Combination enhances the degree of caspase-3 release as compared to the alone drugs and release of caspase-3 was also significantly increased from 12 hrs to 72 hrs of treatment as shown in Figure 15 and Figure 16. The combination of Cisplatin and RITA significantly increased the caspase-3 release as compared with control, as well as their individual effect at 5µM and 10µM concentration . Whereas increasing the concentration of cisplatin and RITA (10µM), did not affect much to caspase-3 release and was found to be equally effective to that of low dose (5µM) concentration

**Table 1:** Effect of Various Doses of Cisplatin and RITA, Alone and in Combination on Percentage Cell Viability in COLO-205 and PC-3 Cells

Treatment and dose	% cell viability (Mean ± S.D.)	
	COLO-205	PC-3
Control	100 ± 0	100 ± 0
Cisplatin 5µM	65.8 ± 2.46 ^a^	66.8 ± 3.23 ^a^
Cisplatin 10µM	58.03 ± 2.20 ^a,b^	59.0 ± 3.27 ^a^
RITA 5µM	58.60 ± 2.00 ^a^	52.73 ± 5.90 ^a,b^
RITA 10µM	52.43 ± 2.67 ^a,c^	36.53 ± 2.58 ^a,c^
Combination of cisplatin and RITA 5+5µM	27.23 ± 5.43 ^d^	24.9 ± 5.01 ^d^
Combination of cisplatin and RITA 10+10µM	20.03 ± 2.85 ^e^	18.56 ± 1.5 ^e^

Values are expressed as Mean **±**S.D. ^a^ p<0.05 vs. control, ^b^ p<0.05 vs. cisplatin 5µM, ^c^ p<0.05 vs. cisplatin 10µM, ^d^ p<0.05 vs. RITA 5µM, ^e^p<0.05 vs. RITA 10µM.

**Table 2:** Effect of Various Doses of Cisplatin and RITA, Alone and in Combination on Chemosensitization in COLO-205 and PC-3 Cells

Treatment	No. of colonies (Mean ± S.D.)	
	COLO-205	PC-3
Control	44.0 ± 2.0	30.6 ± 1.52
Cisplatin 5µM	23.0 ± 1.0 ^a^	20.33 ± 1.15 ^a^
Cisplatin 10µM	16.3 ± 1.52 ^a,b^	14.66 ± 1.52 ^a,b^
RITA 5µM	15.0 ± 2.0 ^a^	16.0 ± 2.00 ^a^
RITA 10µM	11.0 ± 1.0 ^a,c^	9.66 ± 1.52 ^a,c^
Combination of cisplatin and RITA 5+5µM	7.0 ± 1.0 ^d^	7.66 ± 1.52 ^d^
Combination of cisplatin and RITA 10+10µM	3.33 ±1.52 ^e,f^	4.00 ±1.00 ^e^

Values are expressed as Mean **±**S.D. ^a^ p<0.05 vs. control, ^b^ p<0.05 vs. cisplatin 5µM, ^c^ p<0.05 vs. cisplatin 10µM, ^d^ p<0.05 vs. Cisplatin 5µM and RITA 5µM, ^e^ p<0.05 vs. Cisplatin 10µM and RITA 10µM, *per se* ^f^ p<0.05 vs. d

**Table 3:** Effect of Various Doses of Cisplatin and RITA, Alone and in Combination on Apoptosis on COLO-205 and PC-3 Cells

Treatment	% Apoptotic cells (Mean ± S.D.)	
	COLO-205	PC-3
Control	4.3 ± 2.08	2.66 ± 2.08
Cisplatin 5µM	13.33 ± 1.52 ^a^	10.0 ± 2.0 ^a^
Cisplatin 10µM	22.0 ± 2.0 ^a,b^	18.0 ± 2.0 ^a,b^
RITA 5µM	26.33 ± 3.2 ^a^	20.0 ± 2.0 ^a^
RITA 10µM	32.33 ± 2.51 ^a,c^	28.33 ± 2.51 ^a,c^
Combination of cisplatin and RITA 5+5µM	41.66 ± 1.52 ^d^	35.33 ± 3.05 ^d^
Combination of cisplatin and RITA 10+10µM	47.33 ±2.51 ^e,f^	41 ±1.0 ^e,f^

Values are expressed as Mean **±**S.D. ^a^ p<0.05 vs. control, ^b^ p<0.05 vs. cisplatin 5µM, ^c^ p<0.05 vs. cisplatin 10µM, ^d^ p<0.05 vs. Cisplatin 5µM and RITA 5µM, ^e^ p<0.05 vs. Cisplatin 10µM and RITA 10µM, *per se* ^f^ p<0.05 vs. ^d^ .

**Table 4:** Effect of Various Doses of Cisplatin and RITA, Alone and in Combination on Peak Caspase-3 Activity in COLO-205 and PC-3 Cells 78 hrs after Incubation with Test Drugs

Treatment	% Apoptotic cells (Mean ± S.D.)	
	COLO-205	PC-3
Control	6.1 ± 2.2	6.1 ± 2.2
Cisplatin 5µM	37.66 ± 2.51^a^	38.0 ± 3.5^a^
Cisplatin 10 µM	45.0 ± 3.0^ a,b^	42.0 ± 3.0^a^
RITA 5µM	44.0 ± 3.60^a^	44.6± 2.51^a^
RITA 10µM	55.33 ± 3.51 ^a,c^	56.33 ± 3.51 ^a,c^
Combination cisplatin and RITA 5+5µM	90 ±2.0^d^	88.0 ±3.5^d^
Combination cisplatin and RITA 10+10µM	92.0 ± 3.0^e,f^	92.0 ± 3.0 ^e,f^

Values are expressed as Mean **±**S.D. ^a^ p<0.05 vs. control, ^b^ p<0.05 vs. cisplatin 5µM, ^c^ p<0.05 vs. cisplatin 10µM, ^d^ p<0.05 vs. Cisplatin 5µM and RITA 5µM, ^e^ p<0.05 vs. Cisplatin 10µM and RITA 10µM, *per se* ^f^ p<0.05 vs. ^d^ .

## Discussion

*P53* is a transcription factor that can be activated by a variety of stress signals (Toshiyuki and Reed, 1995). Upon activation, it induces a group of genes necessary to inhibit cell proliferation or induce cell death (Hanahan and Weinberg, 2000). The tumor suppressor *p53* is mutated in a wide variety of human cancers at a frequency of about 50 percent (Vousden and Prives, 2009). It has also been shown that various *MDM2* inhibitors like MI-219 had been found to show synergism with oxiplatin like genotoxic drugs in *wt-p53* solid tumors through the emergence of synergy unique genes (Asfar et al., 2011). Drugs like Nutlins which are known to be potent MDM2 antagonists had shown to exhibit synergism with various genotoxic drugs like Cisplatin in cisplatin resistant testicular carcinoma cells . In support of this notion, we investigated the effect of inhibition of *MDM2-p53* interaction with cisplatin in colon and prostate cancer cell lines; which should lead to p53 mediated cell arrest mechanisms. 

We studied the effect of Cisplatin, RITA and their combination by their in vitro evaluation of their cytotoxic potential and calculated IC_50_. Our results indicate the efficacy of RITA and Cisplatin on colon and prostate cancer cell lines are in agreement with those of previous studies on various malignancies. Moreover, we were able to conclude that the action of Cisplatin was potentiated by RITA. Not only the anti-cancer effect was enhanced, but the genotoxic dose of Cisplatin was also reduced which use to be cytotoxic at higher doses. Colony formation assay showed that treatment with RITA in combination with Cisplatin significantly reduced the number of colonies formed by COLO-205 and *PC-3* cancer cells showing its chemo sensitizing effect. Our results show that combining non-toxic concentrations of RITA (5µM) with cisplatin (at lower doses of 5µM and 10µM) sensitizes intrinsic pathway of apoptosis. Whereas on increasing the concentration of cisplatin and RITA combination (10µM), did not affect much to cell viability and was found to be equally effective to that of low dose (5µM) concentration.

In summary, it can be concluded that with the selective *MDM2-p53* interaction inhibitor RITA, in combination with cisplatin causes a high induction of both p53 and *MDM2*, a massive induction of apoptosis, and a strong reduction in cell survival in colon and Prostate cancer cell lines due to the release on caspase-3 resulting in apoptosis through internal pathway of apoptosis.

Our present study provides a strong evidence that pharmacological activation of the *p53* by blocking the *MDM2–p53* interaction will be promising cancer therapeutic strategy and using RITA in combination with Cisplatin not only decrease the toxic effect of Cisplatin by decreasing its dose but also increasing the apoptotic effect, warrants clinical evaluation as a new cancer therapy by combining *MDM2* inhibitor with the genotoxic drugs like Cisplatin which not only decrease the genotoxic concentration of the drug Cisplatin but also increases its efficacy for apoptosis Though RITA didn’t showed any dose dependent effect, but still it is proving efficacious at lower doses, accounting for its lesser cytotoxicity on normal cells but increasing apoptotic activity through the release of Caspase-3 when used in combination with Cisplatin. However, the involvement of combination in cell cycle regulation as well as increased expression level of* p53* after combination regimen might be investigated by FACS and western blotting techniques respectively, would more clearly depict the role of cell growth inhibition (like apoptosis) in cell cycle regulation, induction of Cell cycle check points, DNA amplification etc.
